# Computational pathology improves risk stratification of a multi-gene assay for early stage ER+ breast cancer

**DOI:** 10.1038/s41523-023-00545-y

**Published:** 2023-05-17

**Authors:** Yuli Chen, Haojia Li, Andrew Janowczyk, Paula Toro, Germán Corredor, Jon Whitney, Cheng Lu, Can F. Koyuncu, Mojgan Mokhtari, Christina Buzzy, Shridar Ganesan, Michael D. Feldman, Pingfu Fu, Haley Corbin, Aparna Harbhajanka, Hannah Gilmore, Lori J. Goldstein, Nancy E. Davidson, Sangeeta Desai, Vani Parmar, Anant Madabhushi

**Affiliations:** 1 Shaanxi Normal University, School of Computer Science, Xi’an, China; 2 Department of Biomedical Engineering, Case Western Reserve University, Cleveland, OH USA; 3 Precision Oncology Center, University of Lausanne, Lausanne, Switzerland; 4 Department of Biomedical Engineering, Georgia Institute of Technology and Emory University, Atlanta, GA USA; 5 Rutgers Cancer Institute of New Jersey, New Brunswick, NJ USA; 6 Perelman School of Medicine, University of Pennsylvania, Philadelphia, PA USA; 7 Department of Population and Quantitative Health Sciences, Case Western Reserve University, School of Medicine, Cleveland, OH USA; 8 University Hospitals Cleveland Medical Center, Cleveland, OH USA; 9 Fox Chase Cancer Center, Philadelphia, PA USA; 10 Fred Hutchinson Cancer Research Center, University of Washington, and Seattle Cancer Care Alliance, Seattle, WA USA; 11 Tata Memorial Centre, Homi Bhabha National Institute, Mumbai, India; 12 Louis Stokes VA Medical Center, Cleveland, OH USA

**Keywords:** Prognostic markers, Breast cancer

## Abstract

Prognostic markers currently utilized in clinical practice for estrogen receptor-positive (ER+) and lymph node-negative (LN−) invasive breast cancer (IBC) patients include the Nottingham grading system and Oncotype Dx (ODx). However, these biomarkers are not always optimal and remain subject to inter-/intra-observer variability and high cost. In this study, we evaluated the association between computationally derived image features from H&E images and disease-free survival (DFS) in ER+ and LN− IBC. H&E images from a total of *n* = 321 patients with ER+ and LN− IBC from three cohorts were employed for this study (Training set: D1 (*n* = 116), Validation sets: D2 (*n* = 121) and D3 (*n* = 84)). A total of 343 features relating to nuclear morphology, mitotic activity, and tubule formation were computationally extracted from each slide image. A Cox regression model (IbRiS) was trained to identify significant predictors of DFS and predict a high/low-risk category using D1 and was validated on independent testing sets D2 and D3 as well as within each ODx risk category. IbRiS was significantly prognostic of DFS with a hazard ratio (HR) of 2.33 (95% confidence interval (95% CI) = 1.02–5.32, *p* = 0.045) on D2 and a HR of 2.94 (95% CI = 1.18–7.35, *p* = 0.0208) on D3. In addition, IbRiS yielded significant risk stratification within high ODx risk categories (D1 + D2: HR = 10.35, 95% CI = 1.20–89.18, *p* = 0.0106; D1: *p* = 0.0238; D2: *p* = 0.0389), potentially providing more granular risk stratification than offered by ODx alone.

## Introduction

Breast cancer is the most frequently diagnosed cancer and the second leading cause of cancer-related death for females worldwide^1,2^. Estrogen receptor-positive (ER+) and lymph node-negative (LN−) is a common subtype of invasive breast cancer (IBC)^3,4^, for which the standard treatment includes the breast-conserving surgery followed by radiation and adjuvant hormonal therapy. The adjuvant chemotherapy is however typically only adopted for the patients in high risk. Given the significant side effects of adjuvant chemotherapy^5,6^, it is critical to identify ER+ and LN− IBC patients with lower ROR who may safely avoid chemotherapy.

Oncotype Dx (ODx)^7–9^ is a widely applied and extensively validated molecular assay in clinical practice with ODx score aiding in estimating the level of recurrence risk of ER+ and LN− IBC and treatment benefit from adjuvant chemotherapy. The ODx test is, however, usually tissue destructive and remains expensive^10^. More importantly, some recent studies^11,12^ have suggested that the ODx assigned risk categories are not always optimal. For example, the test was less accurate on African American patients as compared to Caucasian patients^11^. In addition, some patients identified as in one ODx risk category might actually have opposite ROR^11,12^. The inclusion of additional information or new models that can provide more granular risk stratification within the ODx risk categories could allow for more accurate personalized treatment regimens for women with ER+ and LN− IBC.

The Nottingham grading system (NGS)^13–15^ is routinely used by pathologists to evaluate ROR for ER+ and LN− IBC. The NGS consists of a three-component visual assessment: (1) nuclear pleomorphism referring to variations in nuclear shape, size, and chromatin appearance, (2) mitotic activity relating to tumor cell division and proliferation, and (3) tubule formation reflecting the percentage of tumor cells forming tubule structures. The subjectivity and inter-observer variability however have remained critical challenges for using NGS in clinical practice with rather unsatisfactory concordance highlighted in a number of studies^16–22^.

With the advent of digital pathology, quantitative histomorphometry (QH) has been widely used to quantify tumor morphology from digitized tissue slides to uncover information potentially undiscernible by human vision systems^23–25^. Features related to individual NGS components including nuclear shape variability, mitotic index and ratio of tubule nuclei have been explored in numerous QH studies and validated as associated with risk stratification in breast cancer^26–31^. However, most studies have not performed a comprehensive and simultaneous quantitative analysis of all three NGS components and have not investigated the added prognostic value that QH-based biomarkers could provide over the ODx test.

**Fig. 1:**
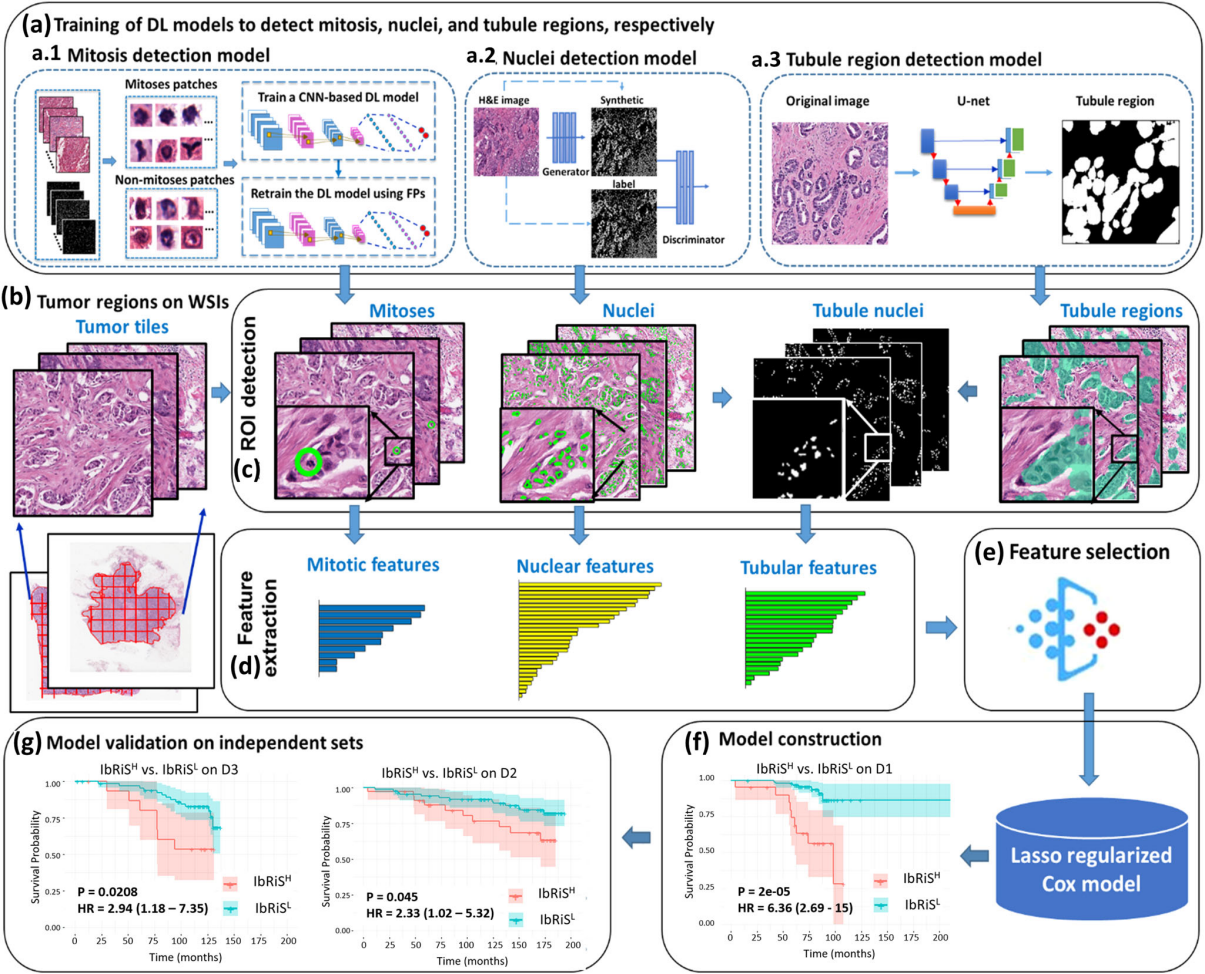
Illustration of the overall workflow for the experimental design. **a** Three deep-learning models: (**a**1) a CNN, (**a**2) a pixel2pixel GAN, and (**a**3) a U-Net model, were trained to detect mitosis, nuclei, and tubules, respectively. **b** Tumor tiles were exhaustively extracted from the tumor regions delineated by an experienced breast pathologist. **c** After detection of mitosis, nuclei, and tubules, quantitative patient-level features were extracted to describe mitotic rates, nuclear pleomorphism, and tubule formation, respectively. **d** The four most prognostic features were selected from each feature category by their association with disease-free survival (DFS) using a Cox regression model. **e** The top features identified from individual feature families were ensembled to train a final combined Cox proportional hazards model to stratify the ER+ and LN− breast cancer patients into high- and low-risk categories on the training set D1 with group differences assessed by two-sided log-rank test. **f** The prognostic model was subsequently locked down (**g**) and evaluated on two independent validation cohorts, D2 and D3 with the differences between high- and low-risk categories measured by two-sided log-rank test.

In this study, we hypothesize that integrating all three components using QH analysis will improve breast cancer prognosis for clinical decision making. In this work, we present an Image-based Risk Score (IbRiS) classifier that combines computer-extracted features of nuclear morphology, mitotic rates, and tubule formation to prognosticate outcomes for ER+ and LN− IBC. The overall workflow is shown in Fig. 1. First, we trained three different deep learning models on H&E-stained Whole Slide Images (WSI) of breast cancer, namely (a1) a Generative Adversarial Network (GAN) for nuclei segmentation, (a2) a deep Convolutional Neural Network (CNN) for mitosis detection, and (a3) a U-Net model for tubule segmentation. Second, based on these computationally derived segmentation/detection masks, we extracted a total of 343 QH features related to nuclear morphology, mitotic count, and tubule formation from the tumor region. Subsequently, we identified the top four prognostic features from each of the three feature categories using a Cox proportional hazards regression model^32^. The top identified features were further ensembled to construct a final prognostic Cox regression model (IbRiS) by associating them with patient clinical outcomes. Finally, we independently validated the prognostic significance of IbRiS on two cohorts from two different institutions, comprising a total of 205 patients with ER+ and LN− IBC. Given the diverse representation of race, tumor grade, and treatment regimen between the training and testing sets, we sought to demonstrate the generalizability of IbRiS for assessing the aggressiveness of breast cancer using computer-extracted histologic features. The prognostic performance of IbRiS was also evaluated within each ODx derived risk category (i.e., low, intermediate, and high).

## Results

### Clinicopathological variables of the patient cohorts

The clinicopathological variables and clinical outcomes of patient cohorts D1, D2, and D3 are provided in Table 1. Patients were primarily in their 50s and 60s, and multiple ethnicity groups were included (non-Hispanic white: 62.6%, South Asian: 26.2%, non-Hispanic black: 9%, other: 2.2%). Notably, unlike the non-Hispanic-white-dominated training set D1 and the validation set D2, all patients in the D3 validation set were South Asian women. Approximately 82% of the patients in D1, D2, and D3 were diagnosed as histologic grade 2/grade 3. Particularly, 63% of the patients in D3 were grade 3, much higher than the 16% in D1 and the 27.3% in D2. The vast majority of the patients in D1 and D3 were HER2 negative (HER2−) (except one HER2 positive (HER2+) case in D1) while in D2, 42% patients were HER2−, 20% patients were HER2+, and 38% had unknown HER2 status. Additionally, 65% of all the patients in D_1 + 2 + 3_ (D1 + D2 + D3) were treated with adjuvant chemotherapy (28% in D1, 100% in D2, and 68% in D3). Of note in D1, chemotherapy use was likely guided by the ODX score, unlike the other two cohorts.

**Table 1:** Summary of clinicopathological variables of the three patient cohorts.

Clinical variables	Training set D1 (UH) *N*(%)	Validation set D2 (ECOG) *N*(%)	Validation set D3 (TMC) *N*(%)
No of patients	116	121	84
Age	59.7 ± 10.4	50.0 ± 8.8	50.4 ± 10.4
≥50 years	99(85%)	63(52%)	40(48%)
<50 years	17(15%)	58(48%)	44(52%)
Race			
Non-Hispanic white	89(77%)	112(93%)	0(0%)
Non-Hispanic black	26(22%)	3(2%)	0(0%)
South Asian	0(0%)	0(0%)	84(100%)
Other	1(1%)	6(5%)	0(0%)
PR status			
Positive	98(84%)	105(87%)	72(86%)
Negative	17(15%)	16(13%)	12(14%)
Unknown	1(1%)	0(0%)	0(0%)
HER2 status			
Positive	1(0.9%)	24(20%)	0(0%)
Negative	112(96.5%)	51(42%)	84(100%)
Unknown	3(2.6%)	46(38%)	0(0%)
Histologic grade			
Grade 1	26(22%)	15(12.4%)	1(1%)
Grade 2	72(62%)	57(47.1%)	30(36%)
Grade 3	18(16%)	33(27.3%)	53(63%)
Unknown	0(0%)	16(13.2%)	0(0%)
Tumor size			
≤20 mm	75(65%)	65(54%)	17(20%)
>20 mm	40(34%)	55(45%)	60(72%)
Unknown	1(1%)	1(1%)	7(8%)
Chemotherapy			
Yes	32(28%)	121(100%)	57(68%)
No	82(71%)	0(0%)	6(7%)
Unknown	2(1%)	0(0%)	21(25%)
Event status			
Event	22(19%)	23(19%)	21(25%)
Censored	90(78%)	98(81%)	51(61%)
Unknown	4(3%)	0(0%)	12(14%)

### Experiment 1: model construction and validation

**Fig. 2:**
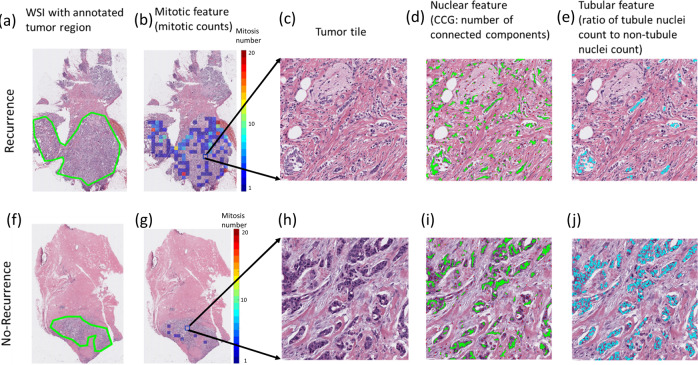
Representative H&E WSI comparison of a recurrent (top row) and a censored (bottom row) patient. The first column (**a**, **f**) shows the original WSI with the pathologist-annotated tumor region. The second column (**b**, **g**) illustrates the distribution of mitotic counts on the WSI with warmer color in the scale bar indicating a higher mitosis number. The third column (**c**, **h**) is a magnified view of a tumor tile. The fourth column (**d**, **i**) demonstrates the top-identified nuclear feature, which quantifies the number of connected nuclei clusters (connected in green line). The fifth column (**e**, **j**) shows the tubule feature “ratio of tubule nuclei count to non-tubule nuclei count” with tubule nuclei highlighted in cyan.

A total of 12 prognostic features were obtained by combining the top 4 features identified in each of the three feature categories (i.e., nuclear morphology, mitotic rates, and tubule formation) using a Cox regression model targeting DFS on D1 (see Supplementary Table 1 for a brief description of the 12 identified top features). The distribution of the four identified features from each of the nuclear, mitotic, and tubule feature categories between the high-risk and low-risk groups predicted by IbRiS on all cohorts D_1+2+3_ is illustrated in Supplementary Fig. 2. Three representative features (i.e., mitotic counts, locally connected nuclear clusters, and the ratio of tubule nuclei count to non-tubule nuclei count) are presented in Fig. 2. Figure 2 shows that patients who did not have DFS events tended to have fewer mitotic events, fewer connected nuclear clusters, and a higher proportion of tubule nuclei in relation to those patients who did experience an event.

**Fig. 3:**
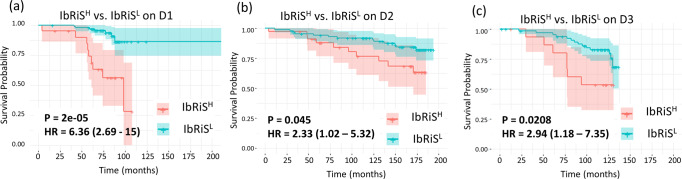
Prognostic performance of IbRiS on D1-D3. KM curve estimates for DFS for IbRiS^H^ (red) versus IbRiS^L^ (blue) across D1–D3 (**a**–**c**), with IbRiS^H^ demonstrating a significantly worse prognosis compared to IbRiS^L^ on D1, D2, and D3 using two-sided log-rank approach.

A LASSO regularized Cox regression model (IbRiS) was constructed with the 12 identified features correlating to DFS on D1 (*n* = 116) (see Supplementary Table 2 for the non-zero coefficients of the features). A dichotomized risk category was generated from the model as described in the “Results” section. The distribution of the continuous risk scores for each individual cohort is illustrated in Supplementary Fig. 3. KM survival curves were generated for high (IbRiS^H^) and low (IbRiS^L^) risk groups for datasets D1, D2, and D3, respectively, with hazard ratio (HR) = 6.36 (95% confidence interval (CI) = 2.69–15, *p* = 2 × 10^–5^) on D1, HR = 2.33 (95% CI = 1.02–5.32, *p* = 0.045) on D2, and HR = 2.94 (95% CI = 1.18–7.35, *p* = 0.0208) on D3 (see Fig. 3). Patients predicted as high risk by IbRiS had a significantly worse outcome in terms of DFS than patients in the low-risk group. Notably, the separation of KM curves between IbRiS^H^ and IbRiS^L^ risk groups was more evident beyond the early survival times (~50 months), which reveals the model’s capability in identifying late DFS events. Since 20% of patients in D2 were HER2 positive and 38% had unknown HER2 status, we additionally performed survival analysis of IbRiS on HER2− patients in D2 after excluding the patients with HER2+ or unknown HER2 status (1st plot in Supplementary Fig. 4) as well as on HER2− and HER2 unknown patients in D2 after excluding patients with HER2+ status (2nd plot in Supplementary Fig. 4). In both KM curves, the trend that the IbRiS^H^ group had a poorer outcome in terms of DFS was observed, although the survival differentiation is not statistically significant, potentially due to the low number of patients included.

Univariate and multivariable Cox proportional hazards analyses for DFS on IbRiS-derived risk category, clinicopathological variables, chemotherapy treatment, and ODx risk category on D1, D2, and D3 are shown in Table 2. On univariate analysis, except for IbRiS-derived risk categories and age on D1, none of the clinicopathological factors was significantly prognostic of DFS on D1, D2, and D3. The patients in IbRiS^H^ had significantly worse DFS compared to those in IbRiS^L^ with HR = 6.36 (95% CI = 2.69–15, *p* = 2e−05) on D1, HR = 2.33 (95% CI = 1.02–5.32, *p* = 0.0450) on D2, HR = 2.94 (95% CI = 1.18–7.35, *p* = 0.0208) on D3). The ODx risk category was significantly prognostic on D1 (HR = 2.48, 95% CI = 1–6.2, *p* = 0.0497) and D2 (HR = 14, 95% CI = 1.74–110, *p* = 0.0132) when combining the intermediate and high-risk category into a single group. In multivariable analysis, IbRiS was found to be independently prognostic of DFS in the training set and both independent testing sets with HR = 6.05 (95% CI = 2.33–16, *p* = 0.0002) on D1, HR = 4.51 (95% CI = 1.1–18, *p* = 0.0366) on D2, and HR = 4.12 (95% CI = 1.45–12, *p* = 0.0078) on D3. Note that we excluded the ODx risk category from the multivariable analysis on D2 due to the limited number of patients with ODx scores (23% in D2) available. In order to investigate the interdependency between IbRiS and ODx risk category on D2, Lin’s concordance correlation coefficient^33^ was calculated with the value of 1 indicating a perfect agreement and −1 representing completely disagreement. The concordance was found to be low between IbRiS (low versus high-risk group) and ODx test (low and intermediate versus high ODx risk category: 0.16 (95% CI = −0.21–0.49); low versus intermediate and high ODx risk category: 0.26 (95% CI = −0.08–0.54)).

**Table 2:** Univariate and multivariable analysis for DFS on IbRiS-derived risk category, clinicopathological variables, chemotherapy treatment, and ODx risk category on D1, D2, and D3.

Univariate analysis			
Clinical variables	UH (D1) Hazard ratio (95% confidence interval, *p*), patient number	ECOG 2197 (D2) Hazard ratio (95% confidence interval, *p*), patient number	TMC (D3) Hazard ratio (95% confidence interval, p), patient number
Age (<50 years vs. ≥50 years)	HR = 0.33 (95% CI = 0.12–0.85, *p* = 0.0218), *N* = 116	HR = 0.85 (95% CI = 0.37–1.9, *p* = 0.6927), *N* = 121	HR = 0.97 (95% CI = 0.41–2.3, *p* = 0.9426), *N* = 84
Race (Non-Hispanic white vs. Others)	HR = 0.55 (95% CI = 0.22–1.4, *p* = 0.1918), *N* = 116	N/A	N/A
Tumor size (<20 mm vs. ≥20 mm)	HR = 0.93 (95% CI = 0.37–2.3, *p* = 0.8715), *N* = 115	HR = 1.47 (95% CI = 0.64–3.4, *p* = 0.3588), *N* = 120	HR = 0.93 (95% CI = 0.31–2.8, *p* = 0.8944), *N* = 77
PR (PR− vs. PR+)	HR = 3.53 (95% CI = 0.47–26, *p* = 0.2185), *N* = 115	HR = 1.66 (95% CI = 0.39–7.1, *p* = 0.4953), *N* = 121	HR = 1.57 (95% CI = 0.37–6.7, *p* = 0.545), *N* = 84
HER2 (HER2− vs. HER2+)	HR = 2.5642 (95% CI = 0.02–18.80, *p* = 0.5678), *N* = 113	HR = 0.79 (95% CI = 0.25–2.5, *p* = 0.6843), *N* = 75	N/A
Histologic grade (high vs. intermediate vs. low)	HR = 1.92 (95% CI = 0.94–3.9, *p* = 0.0725), *N* = 116	HR = 1.58 (95% CI = 0.77–3.2, *p* = 0.216), *N* = 105	HR = 0.85 (95% CI = 0.36–2, *p* = 0.7084), *N* = 84
Histologic grade (high and intermediate vs. low)	HR = 1.83 (95% CI = 0.54–6.2, *p* = 0.333), *N* = 116	HR = 6.89 (95% CI = 0.94–877.43, *p* = 0.0599), *N* = 105	N/A
Histologic grade (high vs. intermediate and low)	HR = 2.48 (95% CI = 0.96–6.4, *p* = 0.0609), *N* = 116	HR = 1.21 (95% CI = 0.47–3.1, *p* = 0.6945), *N* = 105	HR = 0.85 (95% CI = 0.36–2, *p* = 0.7084), *N* = 84
Chemotherapy (yes vs. no)	HR = 1.28 (95% CI = 0.52–3.2, *p* = 0.5909), *N* = 114	N/A	HR = 0.56 (95% CI = 0.16–1.9, *p* = 0.3623), *N* = 63
Oncotype Dx (ODx) category (high vs. intermediate vs. low)	HR = 1.85 (95% CI = 1.05–3.3, *p* = 0.0348), *N* = 116	HR = 3.07 (95% CI = 1.36–6.9, *p* = 0.0067), *N* = 28	N/A
Oncotype Dx (ODx) category (high and intermediate vs. low)	HR = 2.48 (95% CI = 1–6.2, *p* = 0.0497), *N* = 116	HR = 14 (95% CI = 1.74–110, *p* = 0.0132), *N* = 28	N/A
Oncotype Dx (ODx) category (high vs. intermediate and low)	HR = 2.18 (95% CI = 0.73–6.5, *p* = 0.1608), *N* = 116	HR = 3.32 (95% CI = 0.82–13, *p* = 0.0937), *N* = 28	N/A
Model derived risk category (IbRiS^H^ vs. IbRiS^L^)	HR = 6.36 (95% CI = 2.69–15, *p* = 2e−05), *N* = 116	HR = 2.33 (95% CI = 1.02–5.32, *p* = 0.045), *N* = 121	HR = 2.94 (95% CI = 1.18–7.35, *p* = 0.0208), *N* = 84

Multivariable analysis			
Clinical variables	UH (*N* = 113)	ECOG 2197 (*N* = 65)	TMC (*N* = 58)
Age (<50 years vs. ≥50 years)	HR = 0.66 (95% CI = 0.22–2, *p* = 0.4739)	HR = 1.75 (95% CI = 0.47–6.5, *p* = 0.4023)	HR = 1.03 (95% CI = 0.37–2.9, *p* = 0.9515)
Race (Non-Hispanic white vs. Others)	HR = 0.69 (95% CI = 0.26–1.9, *p* = 0.4614)	N/A	N/A
Tumor size (<20 mm vs. ≥20 mm)	HR = 0.8 (95% CI = 0.28–2.3, *p* = 0.6732)	HR = 2.33 (95% CI = 0.54–10, *p* = 0.2565)	HR = 1.15 (95% CI = 0.37–3.6, *p* = 0.8133)
PR (PR− vs. PR+)	HR = 2.58 (95% CI = 0.34–20, *p* = 0.3611)	HR = 0.41 (95% CI = 0.07–2.3, *p* = 0.3124)	HR = 2.72 (95% CI = 0.32–23, *p* = 0.3564)
HER2 (HER2− vs. HER2+)	HR = 1.15 (95% CI = 0.01–24.28, *p* = 0.9340)	HR = 0.89 (95% CI = 0.24–3.4, *p* = 0.8688)	N/A
Histologic grade (high vs. intermediate vs. low)	HR = 1.36 (95% CI = 0.6–3.1, *p* = 0.4591)	HR = 1.32 (95% CI = 0.48–3.7, *p* = 0.5921)	HR = 1.01 (95% CI = 0.35–2.9, *p* = 0.9894)
Oncotype Dx (ODx) category (high vs. intermediate vs. low)	HR = 2.11 (95% CI = 0.97–4.6, *p* = 0.0606)	N/A	N/A
Model derived risk category (IbRiS^H^ vs. IbRiS^L^)	HR = 6.05 (95% CI = 2.33–16, *p* = 0.0002)	HR = 4.51 (95% CI = 1.1–18, *p* = 0.0366)	HR = 4.12 (95% CI = 1.45–12, *p* = 0.0078)

### Experiment 2: IbRiS-derived risk category versus ODx risk category

**Fig. 4:**
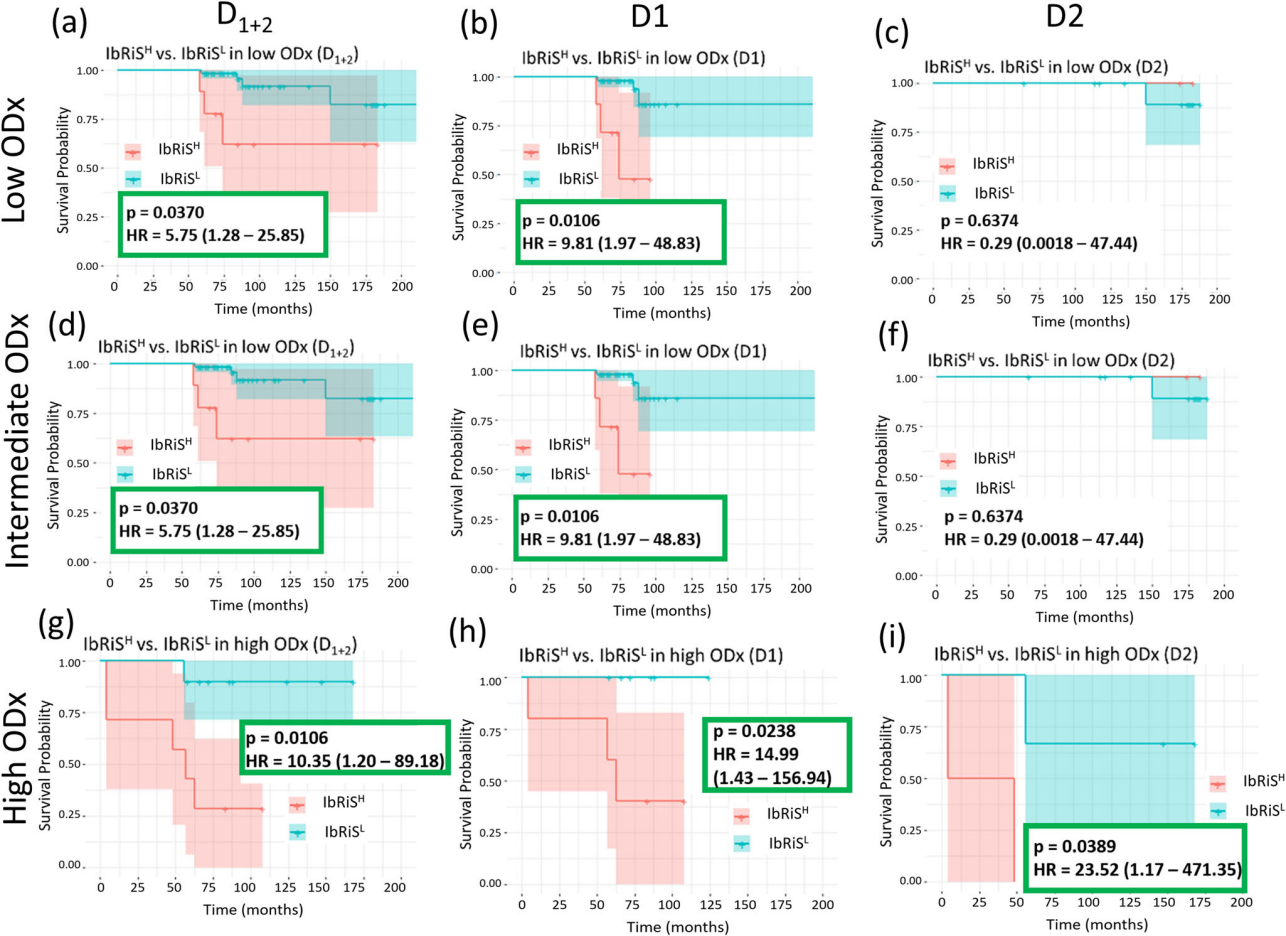
Prognostic performance of IbRiS within individual ODx risk category in D1-D2. KM curve estimates for DFS for IbRiS^H^ (red) versus IbRiS^L^ (blue) in the low, intermediate, and high ODx risk categories, respectively across D_1+2_ (**a**, **d**, **g**), D1 (**b**, **e**, **h**) and D2 (**c**, **f**, **i**) with the differences between the risk categories assessed by two-sided log-rank test. IbRiS was significantly prognostic within high ODx risk category for both D1 and D2.

We sought to demonstrate the prognostic ability of IbRiS-derived risk scores within each individual ODx category. ODx scores were available for *n* = 116 patients in D1 and *n* = 28 patients in D2. As shown in the KM curves in Fig. 4, patients in the IbRiS^H^ group experienced a higher relapse probability than those classified as IbRiS^L^ in the high ODx categories for both D1 and D2. Specifically, in the high ODx risk category (D_1+2_), among the 10 patients predicted as IbRiS^L^, 9 patients had favorable outcomes (non-DFS event with a median follow-up of ~7 years) while among the 7 patients identified as high risk by IbRiS, 5 of them suffered recurrence/death.

We additionally generated KM plots for DFS for the low versus intermediate versus high ODx risk categories to demonstrate the prognostic performance of ODx risk category on D1 and D2, as shown in Supplementary Fig. 5.

### Experiment 3: IbRiS-derived risk category versus histologic grade

**Fig. 5:**
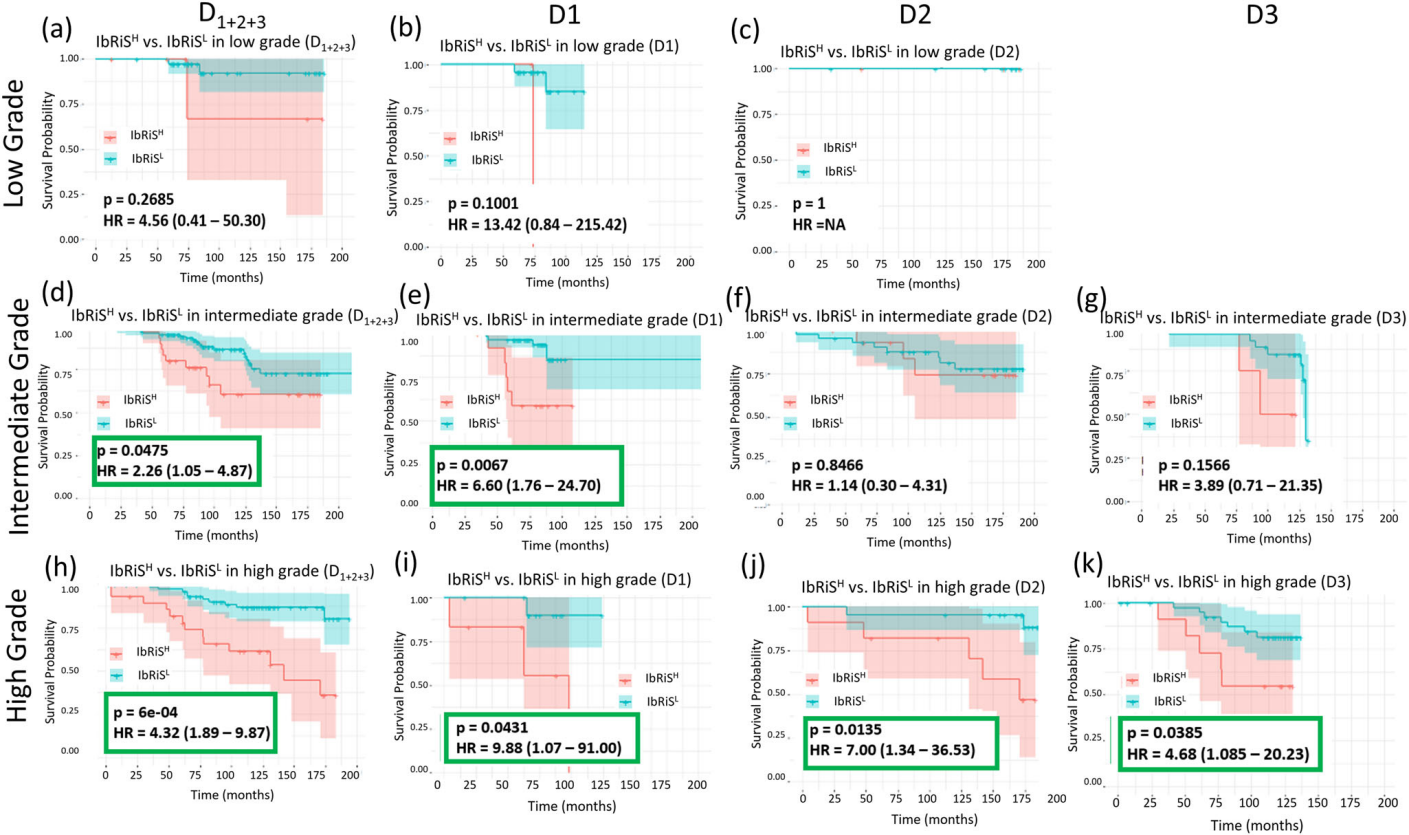
Prognostic performance of IbRiS within individual histology grade in D1-D3. KM curve estimates for DFS for IbRiS^H^ (red) versus IbRiS^L^ (blue) in the low, intermediate, and high histologic grades, respectively across D_1+2+3_ (**a**, **d**, **h**), D1 (**b**, **e**, **i**), D2 (**c**, **f**, **j**) and D3 (**g**, **k**) with the differences between the risk categories assessed by two-sided log-rank test. IbRiS was significantly prognostic within high histologic grade groups for all three cohorts.

We sought to demonstrate the prognostic ability of IbRiS-derived risk categories in subgroups stratified by pathologist-assigned histologic grades. As shown in the KM curves in Fig. 5, for the high-grade groups, patients predicted as IbRiS^H^ had significantly worse prognosis than those predicted as IbRiS^L^ for all the three cohorts. Specifically, for the pathologist-assigned high-grade group (D_1+2+3_), 50% of patients identified as IbRiS^H^ suffered from DFS events, while among the patients classified as IbRiS^L^ only 14% recurred/died.

We additionally generated KM plots for DFS for the low versus intermediate versus high histologic grade groups to demonstrate the prognostic performance of histologic grade on D1, D2 and D3 as shown in Supplementary Fig. 5. The survival analysis of clinical risks (simultaneously considering tumor grade and tumor size)^34^ in terms of DFS was also performed on the combination of three cohorts (D_1+2+3_) as shown in Supplementary Fig. 6.

## Discussion

Oncotype Dx (ODx)^8,9^ is a widely applied multi-gene-based assay in clinical practice that has been clinically validated to be prognostic and predictive of treatment benefit of adjuvant chemotherapy. However, ODx is expensive and tissue destructive. More importantly, consistent disagreement in risk classification between the ODx test and other molecular assays has been identified, with ODx incorrectly identifying a number of patients who are likely to have a low risk of recurrence as high risk. In one comparison study^12^ between ODx and Prosigna (another FDA approved Prognostic Gene Signature), Prosigna was found to be a better indicator of ROR than the ODx test. In addition, ODx was found to be significantly less accurate in African American versus Caucasian breast cancer patients, suggesting ODx was not well calibrated for racial/ethnic minority populations^11^.

In this study, we presented a digital-pathology-based classifier to risk stratify ER+ and LN− breast cancer patients by comprehensively measuring characteristics related to the nuclear histomorphology, tubule formation, and mitotic activity from H&E-stained slide images. Additionally, we investigated if the image risk model (IbRiS) was able to provide further granular prognostic value over the ODx test. While a few studies have shown the association between QH features extracted from digitized H&E-stained slides and the ODx risk categories^30,31,35,36^, these studies either solely focused on one component of the three feature categories (i.e., nuclear morphology, mitotic rates, and tubule formation) or did not explore the added prognostic value the image-based signatures could offer over the ODx test. For example, Whitney et al.^35,36^ assessed the ability of computerized nuclear shape and architecture features to predict ODx risk categories for breast cancer patients. Romo-Bucheli et al.^30^ developed a deep learning based mitosis detection classifier on WSIs and further evaluated the correlation of mitosis count with ODx risk categories for breast cancer patients.

In our study, from the survival analysis of IbRiS in the subgroups of ODx risk categories, we found that IbRiS was able to add significant prognostic value to the ODx risk category (Fig. 4). For the patients distributed in the high ODx category, IbRiS was able to identify patients with true low ROR. These results suggest that among the patients identified as high risk by ODx test in clinical practice, some of them, however, are in fact true low risk and could be effectively identified by IbRiS, thus safely avoiding aggressive adjuvant chemotherapy.

IbRiS was validated as prognostic on two independent validation cohorts independent of clinicopathological variables. In addition, we performed survival analysis using histologic grade on D1, D2 and D3 and ODx risk category on D1 and D2 (Supplementary Fig. 5). Notably, the rate of chemotherapy administration varied among the subgroups in D1 with 26.6% for IbRiS^L^ versus 35% for IbRiS^H^, 1.15% for the low versus 23.9% for the intermediate versus 70.6% for the high histologic grade, and 6.67% for low versus 42.9% for intermediate versus 83% for high ODx risk category. The heterogeneity in treatment among the subgroups (in terms of IbRiS-derived risk groups, histologic grades, or ODx risk categories) resulted in a differing impact on the corresponding patient outcome. In the scenario of homogeneous therapy, with higher survival improvement due to higher chemotherapy administration rate in the high-risk group could being effectively eliminated, the risk stratification among the risk groups could be potentially increased in D1.

The Nottingham Grading Scheme (NGS) is one of the most commonly utilized traditional prognostic factors for IBC by pathologists in routine clinical practice^13,14^. The NGS includes the measurements of nuclear pleomorphism, mitotic count, and tubular differentiation to assess tumor aggressiveness and stratify breast cancer patients by ROR. While not significantly prognostic, histologic grades still showed a certain level of prognostic value in our study, as evidenced by the marginal significance in univariable analysis in D1 and D2 (D1: *p* = 0.0609 for high versus intermediate and low grade; D2: *p* = 0.0599 for high and intermediate versus low grade) and survival analysis for D_1+2+3_ in Supplementary Fig. 5. A possible reason for the non-significant prognostic value of histologic grade could be the relatively small number of patients and DFS events in the cohorts considered. However, the poor to moderate inter-reader disagreement with breast cancer grading has remain a critical challenge in pathology practice^16–22^. For example, in an ECOG study of inter-observer reproducibility of NGS in stage II breast cancer, two committee pathologists only concurred on the histologic grade for 54% of patients, marginally higher than the agreement rate expected by chance^16^. Taking this into account, it is imperative to develop an objective and accurate prognostic model as a complementary tool to NGS in clinical practice to improve the assessment of ROR for breast cancer patients. For example, Wang et al.^37^ built DeepGrade, a CNN-based model for further risk stratification of the breast tumors in intermediate histology grade. DeepGrade was trained for binary classification of low versus high histological grade with H&E WSIs of breast tumors. DeepGrade was then applied to re-stratify the tumors with intermediate histologic grade into high- (similar with high-grade) or low- (similar with low grade) risk groups, with the predicted high-risk group showing a significantly elevated ROR compared with the low-risk group. Similarly, Jaroensri et al.^38^ constructed three DL models to predict pathologist-based reference standards for the three NGS components, respectively. They also found that the AI-NGS combining the output of the three DL models delivered non-inferiority performance for breast cancer prognosis compared with pathologists grading. Our study differs from these two studies in a couple of critical ways: First, the abstract image representations captured in the two studies for model predictions lack biologic interpretability as compared with the biologically explainable QH features employed in our study. Second, DeepGrade and AI-NGS were both trained with histologic grade as ground truth and the models’ prognostic significance were then demonstrated. In contrast, in our study, IbRiS was constructed by directly associating the image features with survival outcome. Additionally, the prognostic relevance of the IbRiS classifier was investigated in the context of all three histologic grade groups. As shown in Fig. 5, IbRiS significantly stratified the high- and low-risk patients within the high histologic groups. These results suggest that with the added prognostic value of IbRiS to histologic grade, the patients at true low risk could be further distinguished from the high-grade group who could potentially safely omit the adjuvant chemotherapy.

The potential clinical impact of IbRiS lies in complementing the ODx test and histologic grade in clinical practice for a more accurate assessment of ROR for breast cancer patients. In some ways, IbRiS more closely mirrors a multi-gene expression-based test like ODx in that it produces a recurrence score based on the weighted combination of the expression of individual and interpretable image features. At least part of the reason for the widespread clinical adoption of ODx has been the inherent interpretability of the test^39^, i.e., being able to connect the recurrence score to the individual genes. Therefore, it stands to reason that IbRiS might be more amenable to clinical adoption than black-box-based deep learning models like DeepGrade. In addition, IbRiS only requires a digital slide image of the biopsy or surgically excised tissue specimen and computing resources to provide the risk score. With the prevalence of WSI scanners, the IbRiS model holds vast potential to serve as an inexpensive and faster alternative prognostic tool in clinical settings, especially in low resource settings where molecular assays like ODx may not be available. Furthermore, IbRiS provides an opportunity to efficiently analyze tumor heterogeneity by processing multiple tissue slides from one tumor and identifying the most relevant features from across the slides for predicting cancer outcomes.

Limitations of our study included the fact that our model was retrospectively validated based on prognostic significance unlike the ODx test, which was prospectively validated for both prognostic significance and treatment benefit prediction. Additionally, our study focused solely on LN− and ER+ IBC patients and had a relatively small sample size. Future work will entail validating the digital pathology-based pipeline in additional independent pan-stage, molecular subtypes, and also in terms of its predictive benefit for adjuvant chemotherapy.

In summary, this study was the first to quantitatively measure the joint QH features of nuclear morphology, mitotic rates, and tubule formation on H&E WSIs and demonstrate its prognostic significance in terms of DFS for ER+ and LN− IBC. In addition, the QH features-based model provided more granular risk stratification within the ODx defined risk category. The prognostic capability of these identified features could also potentially be applicable in IBCs with positive lymph nodes as well as other molecular subtypes.

## Methods

### Dataset description

Our study comprised three independent cohorts (D1, D2, and D3) of patients with ER+ and LN− IBC. H&E-stained slides of surgically resected tumor specimen (no neoadjuvant treatment was administered) from D1, D2, and D3 were respectively digitized using a Roche Ventana iScan HT Scanner, a Philips Scanner, and a Ventana DP 200 Scanner (Hemel Hempstead, UK) at ×40 magnification (0.25 micron per pixel). In our experiments, D1 served as a training set for feature selection and model construction. D2 and D3 served as independent validation sets to evaluate the performance of the final locked-down prognostic model.

The flowchart for the inclusion and exclusion criteria for patient selection on D1, D2 and D3 are shown in Supplementary Fig. 1. A summary of clinicopathological variables of the three cohorts of ER+ and LN− breast cancers is shown in Table 1.

Training cohort D1: breast cancer patients treated between 1996 and 2018 at University Hospitals (UH) having a corresponding ODx score available were identified and retrieved by the pathologists from the hospital archive. The slides were subsequently digitized and transferred. H&E-stained tumor WSI along with clinicopathological/outcome information were available for 519 patients. Patients without any event (recurrence/metastasis/death) were only recruited in this study when at least a 5-year follow-up was available. This process resulted in *n* = 116 ER+ and LN− breast cancer patients (*n* = 22 events) in D1. This study was approved by the Institutional Review Board (IRB) at University Hospitals (IRB No 02-13-42C).

Validation cohort D2: The Eastern Cooperative Oncology Group (ECOG) 2197^40^ is a prospective, randomized, phase III clinical trial that recruited *n* = 2778 patients with IBC (1 to 3 positive LN/LN− with tumor size ≥1 cm) from 1998 to 2007 to compare the patient’s outcome under two different adjuvant chemotherapy regimens (i.e., doxorubicin plus docetaxel versus doxorubicin plus cyclophosphamide; a previous study^40^ identified no significant difference in patient outcomes between the two treatment regimens). ECOG 2197 is deemed an ideal validation set due to the homogeneity in treatment (all the patients received adjuvant chemotherapy), which reduced the effect of treatments on patient outcomes. The access to the ECOG dataset involved a 2-year long process including a proposal review first through ECOG and subsequently through the Cancer Therapy Evaluation Program (CTEP) at the National Cancer Institute (NCI). From this superset, D2 comprises the subset of *n* = 121 ER+ and LN− breast cancer patients (*n* = 23 events), whose corresponding WSIs and clinical information were both accessible. ECOG provided us with the de-identified clinical data from the archived clinical trial along with the de-identified images. This study was approved by the IRB at University Hospitals (IRB No 02-13-42C).

Validation cohort D3: D3 comprises *n* = 84 ER+ and LN− Indian patients treated in 2009 and with follow-up until 2020 (*n* = 21 events) at Tata Memorial Center (TMC) which were identified and retrieved by the pathologist from hospital archive. The H&E stained tumor slides for individual patients were digitized in and subsequently transferred from TMC. The study was approved by Institutional Ethics Committee, TMC, IEC no. 1712.

The study conformed to Health Insurance Portability and Accountability Act (HIPAA) guidelines and was approved by the IRB at University Hospitals (IRB No 02-13-42C). The need for written consent from participants was waived due to the use of de-identified retrospective data.

The tumor region in the WSI was manually annotated or checked by a pathologist, with artifacts intentionally avoided (i.e., tissue folding, pen mark, staining artifacts, and bubbles). The slide with the largest representative tumor was selected for the subsequent analysis for the patients who have multiple slides available.

### Feature extraction

A set of 343 QH features were extracted to describe nuclear morphology, mitotic rates, and tubule formation based on the masks of nuclei, mitosis, and tubules, respectively, generated by three different deep learning models. Additional details regarding deep learning models, algorithms for feature calculation, and descriptions of features extracted are available in the Supplementary Methods. All tiles (2000 × 2000 pixels at ×40 magnification) containing tumor region as annotated by a pathologist were exhaustively extracted from the WSIs. In each individual tile, nuclear morphology, mitotic activity, and tubule formation-related characteristics were computed. The patient-level features were then calculated by aggregating (i.e., mean, median, max, sum, standard deviation, skewness, kurtosis, histogram entropy, and approximate entropy) these features across all the tiles.

Nuclear histomorphometric feature extraction: we employed a Pixel2Pixel GAN for nuclei segmentation. Following nucleus segmentation, we extracted 242 nuclear features to quantify the nuclear histomorphology of each WSI, including global graph^41^, shape, cell cluster graph (CCG)^42^, cell orientation entropy (CORE)^43^, and Haralick texture feature families^44^. Global graph and CCG feature families, respectively, describe the global and local spatial distribution of nuclei; shape features capture nuclear boundary properties such as smoothness and elongation; the CORE feature family quantitatively measures the disorder degree of nuclear orientations; Haralick texture features characterize chromosome patterns within nuclei.

Mitosis feature extraction: a CNN was trained to detect mitotic events on H&E-stained WSIs. In addition, an epithelium segmentation model was trained to identify epithelial nuclei for subsequent mitosis ratio calculation. Forty-five features were extracted from each WSI based on detected mitoses to describe the mitosis prevalence status. More specifically, these features included: (1) multiple statistical measurements of the mitotic count; (2) ratio of mitotic count to epithelial nuclei count, ratio of mitotic count to blue-ratio nuclei count, and ratio of mitotic count to nuclei count, over all of the extracted tumor tiles across the WSI; (3) the proportion of tiles presenting a specific mitotic density within the WSI; and (4) quantitative proliferation score calculated by simulating the mitosis prevalence assessment in clinical practice.

Tubule feature extraction: tubule formation represents the portion of tumor cells forming tubular glands^19^. We trained a U-Net to automatically segment tubules in breast cancer histopathological images. A total of 56 tubule features were extracted to measure tubule formation based on the segmented tubule masks. Those features comprised various statistical summaries of tubule ratio metrics on all the tiles across the WSI of each patient (i.e., the ratio of tubule nuclei count to the non-tubule nuclei count, the ratio of tubule nuclei count to the epithelium nuclei count, and the ratio of tubule nuclei count to the nuclei count) as well as the number of tiles falling between different tubule ratio intervals.

### Feature selection and classifier construction

In total, 343 features were finally extracted (242 nuclear pleomorphism features, 45 mitotic count features, and 56 tubule formation features). A Cox proportional hazards regression model (henceforth referred to as Cox regression model)^32^, regularized by Least Absolute Shrinkage and Selection Operator (LASSO)^45^, was constructed to identify important predictors of DFS. First, to keep the balance among the three feature categories, we implemented a Cox regression model to identify the top four prognostic features associated with DFS separately on each of the three categories on training set D1. The total number of top features (*n* = 12) for inclusion within the model was determined as ~10% of the patient number in the training set. Following feature identification, a final LASSO regularized Cox regression model was used to compute the coefficients for each of the features; 11 features were assigned non-zero coefficients as part of inclusion within IbRiS while one feature had a zero-coefficient value.

An optimal risk score threshold (denoted as “θ_opt_” hereafter) was identified from the training set D1 (see Supplementary Methods for details) to dichotomize the continuous risk scores into binary high/low-risk categories.

### Statistical analysis

IbRiS was validated on two independent testing sets, D2 and D3. Specifically, we calculated a continuous risk score for each patient on D2 and D3 using the feature coefficients estimated from D1. We then classified the patients into a binary high (risk score >θ_opt_) versus low (risk score ≤θ_opt_) risk category of recurrence by applying θ_opt_ identified from D1. DFS was defined as the time from diagnosis/random treatment assignment until first recurrence (loco-regional or distant metastasis) or death, whichever occurred earlier. Patients were censored when they did not have an event at the termination of the study or were lost to follow-up at any time during the study. Kaplan–Meier (KM) survival analysis with DFS as the endpoint was performed between the IbRiS-derived high- versus low-risk categories. The rate of DFS was estimated using the KM method, and the difference of DFS was assessed using log-rank test^46^ between the high- and low-risk categories predicted by IbRiS on D1, D2, and D3. We also performed subgroup survival analysis respectively for high, intermediate, and low ODx risk categories (traditional recurrence score categorization was applied: low: <18, intermediate: 18–30, high: >30)^9^ as well as high, intermediate, and low histologic grades assigned by pathologists.

We conducted a univariate Cox proportional hazard analysis to evaluate if any of the routinely examined clinical parameters, treatments, and ODx risk categories were prognostic of DFS on D1, D2, and D3. The clinical parameters include age (≤50 years versus >50 years), race (white versus other), tumor size (<20 mm versus ≥20 mm), Progesterone Receptor (PR) status (negative versus positive), HER2 status (negative versus positive), histologic grade (Grade I versus Grade 2 versus Grade 3). Multivariable Cox analysis^47^ was also performed to assess the independent prognostic significance of IbRiS after accounting for the other clinicopathological variables on D1, D2, and D3.

### Reporting summary

Further information on research design is available in the Nature Research Reporting Summary linked to this article.

## Supplementary information

Supplementary Materials

Reporting Summary

## Data Availability

The datasets used and/or analyzed during the current study are available from the corresponding author on reasonable request.

## Code availability

The code developed and utilized during the current study are available from the corresponding author on reasonable request.

## References

[1] DeSantis CE (2019). Breast cancer statistics, 2019. CA Cancer J. Clin..

[2] Ibrahim A (2020). Artificial intelligence in digital breast pathology: techniques and applications. Breast.

[3] Siegel RL, Miller KD, Jemal A (2020). Cancer statistics, 2020. CA Cancer J. Clin..

[4] Schootman M, Jeffe D, Reschke A, Aft R (2004). The full potential of breast cancer screening use to reduce mortality has not yet been realized in the United States. Breast Cancer Res. Treat..

[5] Miller KD (2019). Cancer treatment and survivorship statistics, 2019. CA Cancer J. Clin..

[6] Brezden CB, Phillips KA, Abdolell M, Bunston T, Tannock IF (2000). Cognitive function in breast cancer patients receiving adjuvant chemotherapy. J. Clin. Oncol..

[7] Losk K (2020). Oncotype DX testing in node-positive imbreast cancer strongly impacts chemotherapy use at a comprehensive cancer center. Breast Cancer Res. Treat.

[8] Sparano JA (2018). Adjuvant chemotherapy guided by a 21-gene expression assay in breast cancer. N. Engl. J. Med..

[9] Paik S (2004). A multigene assay to predict recurrence of tamoxifen-treated, node-negative breast cancer. N. Engl. J. Med..

[10] Flanagan MB, Dabbs DJ, Brufsky AM, Beriwal S, Bhargava R (2008). Histopathologic variables predict Oncotype DX™ Recurrence Score. Mod. Pathol..

[11] Hoskins KF, Danciu OC, Ko NY, Calip GS (2021). Association of race/ethnicity and the 21-Gene Recurrence Score with breast cancer-specific mortality among US women. JAMA Oncol..

[12] Abdelhakam DA, Hanna H, Nassar A (2021). Oncotype DX and prosigna in breast cancer patients: a comparison study. Cancer Treat. Res. Commun..

[13] Elston CW, Ellis IO (2002). Pathological prognostic factors in breast cancer. I. The value of histological grade in breast cancer: experience from a large study with long-term follow-up. Histopathology.

[14] Saimura M (1999). Prognosis of a series of 763 consecutive node-negative invasive breast cancer patients without adjuvant therapy: analysis of clinicopathological prognostic factor. J. Surg. Oncol..

[15] Rakha EA (2008). Prognostic significance of Nottingham histologic grade in invasive breast carcinoma. J. Clin. Oncol..

[16] Gilchrist KW (1895). Interobserver reproducibility of histopathological features in stage II breast cancer. An ECOG study. Breast Cancer Res. Treat..

[17] van, Dooijeweert C (2020). Significant inter- and intra-laboratory variation in grading of invasive breast cancer: a nationwide study of 33,043 patients in the Netherlands. Int. J. Cancer.

[18] Theissig F, Kunze KD, Haroske G, Meyer W (1990). Histological grading of breast cancer. Interobserver, reproducibility and prognostic significance. Pathol. Res. Pract..

[19] Mansour EG, Ravdin PM, Dressler L (1994). Prognostic factors in early breast carcinoma. Cancer.

[20] Longacre TA (2006). Interobserver agreement and reproducibility in classification of invasive breast carcinoma: an NCI breast cancer family registry study. Mod. Pathol..

[21] Boiesen P (2000). Histologic grading in breast cancer–reproducibility between seven pathologic departments. South Sweden Breast Cancer Group. Acta Oncol..

[22] Jacquemier J, Charpin C (1998). Reproducibility of histoprognostic grades of invasive breast cancer. Ann. Pathol..

[23] Madabhushi A, Lee G (2016). Image analysis and machine learning in digital pathology: challenges and opportunities. Med. Image Anal..

[24] Bhargava R, Madabhushi A (2016). Emerging themes in image informatics and molecular analysis for digital pathology. Annu. Rev. Biomed. Eng..

[25] Bera K, Schalper KA, Rimm DL, Velcheti V, Madabhushi A (2019). Artificial intelligence in digital pathology—new tools for diagnosis and precision oncology. Nat. Rev. Clin. Oncol..

[26] Lu C (2018). Nuclear shape and orientation features from H&E images predict survival in early-stage estrogen receptor-positive breast cancers. Lab. Investig..

[27] Chang JM (2018). Back to basics: traditional Nottingham grade mitotic counts alone are significant in predicting survival in invasive breast carcinoma. Ann. Surg. Oncol..

[28] Jimenez G, Racoceanu D (2019). Deep learning for semantic segmentation vs. classification in computational pathology: application to mitosis analysis in breast cancer grading. Front. Bioeng. Biotechnol..

[29] Chen J-M (2017). Computer-aided prognosis on breast cancer with hematoxylin and eosin histopathology images: a review. Tumor Biol..

[30] Romo-Bucheli D, Janowczyk A, Gilmore H, Romero E, Madabhushi A (2017). A deep learning based strategy for identifying and associating mitotic activity with gene expression derived risk categories in estrogen receptor positive breast cancers. Cytom. Part A: J. Int. Soc. Anal. Cytol..

[31] Romo-Bucheli D, Janowczyk A, Gilmore H, Romero E, Madabhushi A (2016). Automated tubule nuclei quantification and correlation with oncotype DX risk categories in ER+ breast cancer whole slide images. Sci. Rep..

[32] Cox DR (1972). Regression models and life-tables. J. R. Stat. Soc. Ser. B Methodol..

[33] Lawrence IKL (1989). A concordance correlation coefficient to evaluate reproducibility. Biometrics.

[34] Sparano JA (2019). Clinical and genomic risk to guide the use of adjuvant therapy for breast cancer. N. Engl. J. Med..

[35] Whitney J (2018). Quantitative nuclear histomorphometry predicts oncotype DX risk categories for early stage ER+ breast cancer. BMC Cancer.

[36] Whitney J, Janowczyk A, Corredor G, Gilmore H, Madabhushi A (2017). Computer extracted features of nuclear shape and architecture predict oncotype DX risk categories for early stage ER plus breast cancer. Mod. Pathol..

[37] Wang Y (2021). Improved breast cancer histological grading using deep learning. Ann. Oncol.

[38] Jaroensri R (2022). Deep learning models for histologic grading of breast cancer and association with disease prognosis. npj Breast Cancer.

[39] Brewer NT, Richman AR, DeFrank JT, Reyna VF, Carey LA (2012). Improving communication of breast cancer recurrence risk. Breast Cancer Res. Treat..

[40] Goldstein LJ (2005). E2197: phase III AT (doxorubicin/docetaxel) vs. AC (doxorubicin/cyclophosphamide) in the adjuvant treatment of node positive and high risk node negative breast cancer. J. Clin. Oncol..

[41] Aurenhammer F (1991). Voronoi diagrams—a survey of a fundamental geometric data structure. ACM Comput. Surv..

[42] Sahirzeeshan A, Robert V, Jonathan AE, Christhunesa C, Anant M (2013). Cell cluster graph for prediction of biochemical recurrence in prostate cancer patients from tissue microarrays. SPIE, Medical Imaging 2013: Digital Pathology.

[43] Lee, G. et al. Cell orientation entropy (COrE): predicting biochemical recurrence from prostate cancer tissue microarrays. *Medical Image Computing and Computer-Assisted Intervention*—*MICCAI* 396–403 (2013). 10.1007/978-3-642-40760-4_50 24505786

[44] Haralick RM (1979). Statistical and structural approaches to texture. Proc. IEEE.

[45] Tibshirani R (1996). Regression shrinkage and selection via the Lasso. J. R. Stat. Soc. Ser. B Methodol..

[46] Creed J, Gerke T, Berglund A (2020). MatSurv: survival analysis and visualization in MATLAB. J. Open Source Softw.

[47] Cox, D. R. & Oakes, D. *Analysis of Survival Data* (CRC Press, 1984).

