# Supplementary material for: Computational pathology improves risk stratification of a multi-gene assay for early stage ER+ breast cancer
Source: NPJ Breast Cancer. 2023 May 17;9:40. doi: 10.1038/s41523-023-00545-y (PMC10192429; doi:10.1038/s41523-023-00545-y)
Supplement: Supplementary file 1 — Supplementary Materials [file 41523_2023_545_MOESM1_ESM.pdf]

# Supplementary Methods

## Details of Deep Learning Models in the Study

### *Details of Nuclei Segmentation Model:*

Training Data: The training data was a publicly available nuclear annotated dataset from the MoNuSeg grand challenge[1]. The dataset comprised 30 images and around 22,000 carefully annotated nuclear boundaries. The images in this dataset were downloaded from the TCGA archive and comprised tumor slides across seven organs from 18 sites. The inherent diversity of nuclei appearance in this dataset allowed for training a robust model. Image patches corresponding to 256 × 256 pixels were extracted from these images at ×40 magnification and fed into the model during training.

Model Details: The architecture of the nuclei segmentation model in our study is shown in Figure 1 (1b). The Pixel2pixel cGAN[2] is an extension of GAN with both the generator and discriminator being conditioned on auxiliary image information. The cGAN model adopted an end-to-end U-Net network[3] as the generator, a multi-layer convolutional network as the discriminator, both of which were formed based on Convolution-BatchNorm-ReLu[4] modules. Feature matching loss[5] was additionally added in the standard cGAN loss[6] function to improve the stability of model training by regulating the generator to generate data that matched the distribution of the real data. Conditional GAN has been validated as a robust and promising approach for nuclei segmentation task in previous studies[7–9].

### *Details of Tubule Detection Model:*

Training Data: The training dataset comprised image patches corresponding to n=307 early-stage breast cancers that were 2000×2000 pixels in size and at 40x magnification. These image patches were randomly extracted from manually annotated tumor regions on digitized slides in D2 (ECOG 2197). The breast tubule structures in each patch were carefully manually delineated by an experienced pathologist. Patches of size 256x256 pixels were extracted from the images and following data augmentation, a dataset of 1.22×109 (i.e. over 1.2 billion) patches for model training.

Model Details*:* We trained a five-layers end-to-end U-Net to segment tubules in breast cancer histopathological images. The model was implemented with Adam optimizer and built with Convolution-BatchNorm-ReLu[4] modules. The combined edge and class weight-based cross-entropy was employed as the loss function to respectively handle the edge detection and class imbalance issue. Twenty tumor tiles were randomly selected from another 20 WSI, respectively, with tubule masks overlaid on the top for visual evaluation by an experienced pathologist. One of four grades (Excellent, Good, Fair, Poor) was assigned to each tile by the pathologist based on visual examination of machine performance for tubule detection on the tile. The reference accuracy for each category as assigned by the pathologist was:  Excellent: >90%, Good: 80% - 90%, Fair: 70%-80%, Poor: <70%.”. 30% of tiles were ranked as "Excellent", 45% of tiles "Good", and 25% of tiles "Fair".

### *Details of Mitosis Detection Model:*

Training data: The network was trained by a dataset containing 550 annotated mitoses in 311 images in size 2000 x 2000 pixels at 40x magnification from 12 IBC cohorts[10]. Considering the

high inter-observer variability for mitosis annotation[11,12], we had a highly experienced (~20 years) board-certified anatomic pathologist to perform mitosis annotation task to ensure the quality of ground truth. This pathologist has had nearly twenty years of experience in reviewing breast pathology slides and almost ten years of experience in reviewing digital pathology images. Small patches (64x64 pixels) were extracted centering around the mitosis/non-mitosis nuclear centroid as the training set.

Model details: Specifically, the training process of our mitosis detection model consists of patch extraction, model training, and model refining. (*1) Patch Extraction:* We converted the RGB H&E images to gray-scale blue-ratio images, where a higher pixel value indicates higher intensity in blue channel relative to the red and green channels. Only the high blue-ratio pixels, which were evident to capture candidate mitosis, were retained to constitute the training patches for computational efficiency. To address the huge class imbalance in the training set due to the sparsity of positive (mitotic) pixels, we hypo-sampled the negative class by random subsampling and augmented positive class size by expanding each annotated mitotic centroid into a 9-pixel radius circle so as to extract multiple mitotic patches from one single mitosis annotation. (*2) Model Training:* We divided the mitotic image dataset into three subsets: a training subset containing 279 images of size 2000 x 2000 pixels with 499,194 patches of size 64×64 pixels extracted, in which 23.6% were positive and 76.4% negative; a validation subset of 56 images with 127,645 patches; and a test subset consisting of 32 images with 55,092 patches. The model was trained using weighted cross-entropy loss in conjunction with an Adam optimizer with image data augmentation for training size enhancement. The model yielding the highest accuracy on the validation set was selected for further model refining in step 3). *(3) Model Refining*: To further reduce the false-positive detection, we fine-tuned the model with an updated training set by randomly substituting 90% of the negative patches with all the false-positive patches classified by the initially trained model. The optimal refined model was locked down and yielded a balanced accuracy of 0.778 and F1 score of 0.54 on the test set.

### *Details of Epithelium Detection Model:*

Training Data: The training data includes the manual annotations of epithelium on 200 digital pathology images (512x512 pixels) at 10x magnification collected from Johns Hopkins University and Cleveland Clinic Foundation.

Model Details: The model used for epithelium detection was of the same architecture as the nuclear segmentation model described in Section "Details of Nuclei Segmentation Model" and Ten tumor tiles were randomly selected from 10 WSI, respectively, with tubule masks overlaid on the top for visual evaluation by a pathologist. Each tile was assigned one of the four ranking grades (Excellent, Good, Fair, Poor). All tiles were ranked as "Good". The reference accuracy for ranking system is the same with the one used for tubule detection assessment.

## **Detailed Description of the Extracted Computerized Features:**

### *Detailed Description of Nuclear Histomorphometric Features:*

- Nuclear Shape features[13,14] (N=100) capture the information of nuclear boundary such as shape irregularity, which have also been proven to be prognostic for early stage ER+ breast cancer[14].
- Nuclear (Haralick) Texture features[15,16] (N=26) evaluate the heterogeneity patterns relating to the chromatin arrangement within each nucleus.

- Cell Orientation Entropy (CORE) features[17] (N=39) quantitatively measure the disorder of nuclear orientation within local neighborhoods, which have been demonstrated to correlate with recurrence of prostate cancer[17].
- Cell Cluster Graph (CCG) features[18] (N=26) characterize local spatial architecture by constructing sub-graphs on the nuclear nodes in the local tumor neighborhood extracting features such as cell radius, connectivity, and eccentricity. CCG features have been utilized to predict the recurrence risk in prostate cancer[18].
- Global Graph features [19] (N=51) explore nuclear architecture by taking each nucleus as a node and connecting the nodes via Voronoi Diagrams, Delaunay Triangles, and Minimum Spanning Trees. The derived nuclear spatial arrangement and nuclear density measurements have previously shown to be associated with recurrence risk for invasive breast cancer[19] and lung cancer[20].

*Detailed Description of Mitosis Features:*

- Mitosis Count (N=6): The patient-level statistics (mean, median, max, standard deviation, skewness, and kurtosis) were calculated on tile-level mitotic counts for each patient.
- Mitosis Count Ratios (N=25): The patient-level statistics (mean, median, max, standard deviation, skewness, and kurtosis) were calculated on the ratios of mitotic count to nuclei count, blue-ratio nuclei count, and epithelium nuclei count on the tile level, respectively. Moreover, mitotic event, detected nuclei, epithelium nuclei, highlighted blue-ratio nuclei were also accumulatively counted across the WSI to calculate the ratios of the accumulated mitotic count to the other three accumulated counts.
- Mitosis Density Vector (N=13): A mitotic density vector containing 11-dimensional descriptors was constructed. Each bin of the vector calculated the proportion of tiles with n (n ϵ 0, 1 … 9, and n ≥ 10) mitotic events on the WSI, respectively[21]. The histogram entropy and approximate entropy of the mitotic density vector also served as part of the patient-level mitotic features.
- Proliferation Score (N=1): Complying with the clinical criteria for tumor proliferation score assignment, a proliferation score of 1, 2 or 3 was automatically calculated in each WSI. We automated the calculation of the proliferation score on each WSI by simulating the clinical mitosis grading scheme (tumor proliferation score 1 corresponds to a mitotic count of 0-7 per 10 high-power fields (HPFs)[22], score 2 corresponds to 8-15 mitoses count, and score 3 corresponds to ≥16 mitotic count[23,24].). We calculated the patient-level proliferation score feature, as follows:

$$M_{10HPFs} = 2 \times \frac{\sum_{i=1}^{T} M_i}{T} = 2M_{mean} \quad (1)$$

$$\text{fscore}_{mean} = \begin{cases} 1, & M_{10HPFs} \in [0,7] \\ 2, & M_{10HPFs} \in [8,15] \\ 3, & M_{10HPFs} \geq 16 \end{cases} \quad (2)$$

where $M_i$ is the number of mitotic events on the $i$th tile, $T$ is the number of tiles containing mitotic event in a WSI, $M_{mean}$ is the average mitotic count per tile, and $M_{10HPFs}$ is the average mitotic count per 10 HPFs.

*Detailed Description of Tubule Features:*

- Tubule Nucleus Ratios (N=26): Three tubule ratios, including tubule nuclei count to the non-tubule nuclei count, tubule nuclei count to the epithelium nuclei count, and tubule nuclei count to the nuclei count were calculated at tile level. Subsequently, eight statistical summaries (mean, median, max, standard deviation, skewness, kurtosis, histogram

entropy, and approximate entropy) were calculated on the three tile-level features to generate 24 patient-level tubule nucleus ratio features. Total tubule nuclei count, overall nuclei count, and overall epithelial nuclei count in a WSI were also calculated.

- Tubule Ratio Distribution Vector (N=30): A 10-dimensional vector was calculated respectively for the three tile-level tubule ratio features. Each of the vector bins counted the number of tiles with ratio values of 0-5/9, 5/9-10/9, …., 35/9-40/9, 40/9-5, and >5 for the ratio of tubule nuclei count to the non-tubule nuclei count and the ratio of tubule nuclei count to the epithelium nuclei count, ratio values of 0-0.1, 0.1-0.2, …., 0.8-0.9, 0.9-1 for the ratio of tubule nuclei count to the nuclei count.

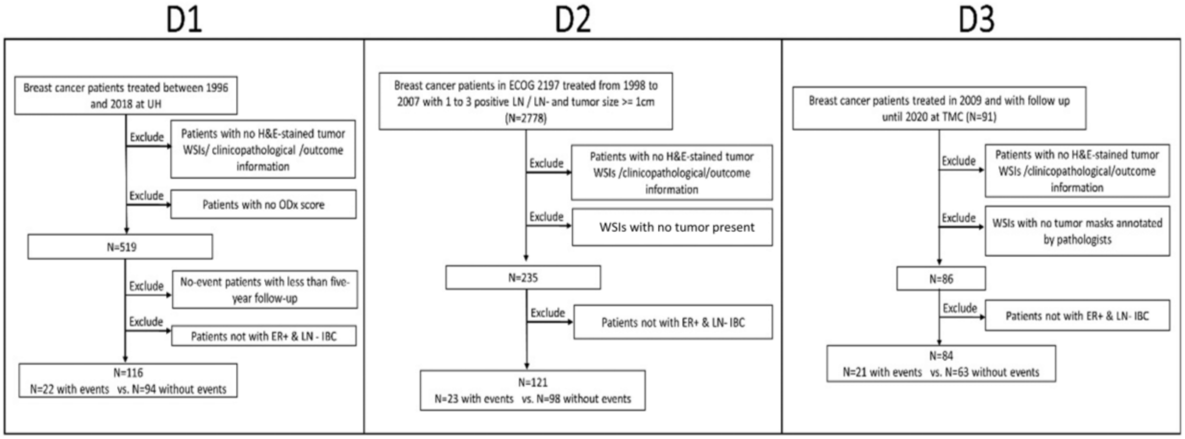


Supplementary figure 1. Flowchart of inclusion and exclusion criteria for patient selection.

| Feature category | Top feature names | Brief description |
|---|---|---|
| Nuclear histomorphometric features | MST: MST Edge Length Minimum/Maximum_mean | Average ratio of maximal to minimal edge length in Minimum Spanning Trees (MST) constructed on nuclei nodes |
| | Shape: Mean Fourier Descriptor6_mean | Average Fourier descriptor 6 of nuclear boundary |
| | CCG: Number of connected components_mean | Average number of cell clusters in the tumor tiles |
| | Haralick: standard deviation intensity information_measure1_mean | Average value of the standard deviation intensity information measure 1 |
| Mitotic features | MtsDensity_n7 | Computerized proliferation score |
| | Proliferation score | Proportion of tiles with 7 mitotic events on the WSI |
| | Overall nuclei number | Overall nuclei number |
| | M2n on WSI | Ratio of mitotic count to the overall nuclei number on a WSI |
| Tubule formation features | TfiDensity_p2_t2nt | Number of tiles with tubule nuclei count to non-tubule nuclei count (t2nt) ratio value of 5/9-10/9 |
| | Kurtosis_t2nt | Kurtosis of tile-level t2nt ratios |
| | Std_t2epi | Standard deviation of tile-level tubule nuclei count to epithelium nuclei count ratios (t2epi) |
| | Max_t2epi | Max values of tile-level t2epi ratios |

Supplementary table 1. A detailed description of the identified 12 top features

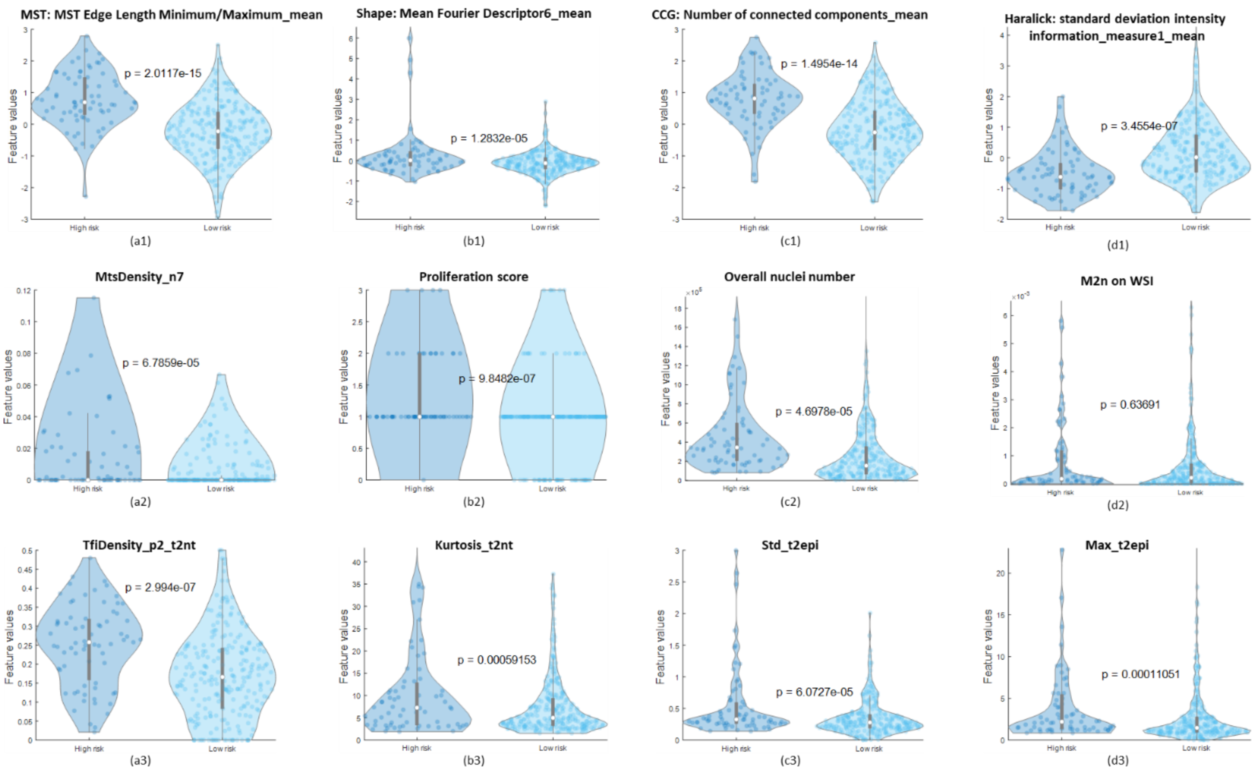


Supplementary figure 2. Illustration of the top 12 identified features between high-risk and low-risk groups predicted by IbRiS on all datasets $D_{1+2+3}$ (D1+D2+D3) with p values calculated by two-sided t-test. The first row corresponds to the top four nuclei features: (a1) MST: MST Edge Length Minimum / Maximum_mean, (b1) Shape: Mean Fourier Descriptor 6_mean, (c1) CCG: Number of connected components mean, and (d1) Haralick: standard deviation intensity information_measure1_mean. The second row shows the top four mitotic features: (a2) MtsDensity_n7, (b2) Proliferation score, (c2) Overall nuclei number, and (d2) M2n_on_wsi. The third row displays the top four tubule features: (a3) TfiDensity_p2_t2nt, (b3) Kurtosis_t2nt, (c3) Std_t2epi, and (d3) Max_t2epi. Among the 12 features, 11 of them were found to be significantly discriminative ($p<0.001$) between the two risk groups.

| **Prognostic feature names** | **Coefficients** | **Feature categories** |
|---|---|---|
| TilesCount_n7_norm | 19.1082 | Mitotic Rates |
| TfiCountNorm_p2_t2nt | 2.9066 | Tubule Formation |
| Std_t2epi | 1.3910 | Tubule Formation |
| Proliferation score _mean | 0.9979 | Mitotic Rates |
| Shape: Mean Fourier Descriptor 6_mean | 0.3637 | Nuclear Morphology |
| MST: MST Edge Length Minimum / Maximum_mean | 0.1951 | Nuclear Morphology |
| CCG: Number of connected components_mean | 0.0746 | Nuclear Morphology |
| Kurtosis_t2nt | 0.0164 | Tubule Formation |
| Overall_nuclei_number | -1.2752e-08 | Mitotic Rates |
| Haralick: standard deviation intensity information_measure1_mean | -0.6235 | Nuclear Morphology |
| M2n_ratio_on_wsi | -255.0056 | Mitotic Rates |

Supplementary table 2. The assigned coefficients corresponding to each of the top features by the prognostic Cox regression model.

## Details of Identification of Optimal Risk Score Threshold

The approach for identification of the optimal risk score threshold in this study has been previously discussed[25] and utilized in another clinical study[26]. Specifically, the optimal threshold to dichotomize the continuous risk scores was adaptably set by a traversal search as follows: The continuous risk scores were first sorted in descending order across all patients in the training set. Subsequently, the average value of each pair of risk scores adjacent to each other was calculated to constitute a set of candidate risk thresholds. The candidate risk-thresholds set was further narrowed by trimming elements with extreme values from both ends. In the traversal search process, every candidate risk threshold was applied to categorize the patients in D1 into high-risk or low-risk recurrence groups with the corresponding log-rank p-value and Hazard Ratio (HR) calculated. The risk-threshold yielding the maximal HR on D1 was selected as the optimal threshold ($\theta_{opt}$) for classifier IbRiS.

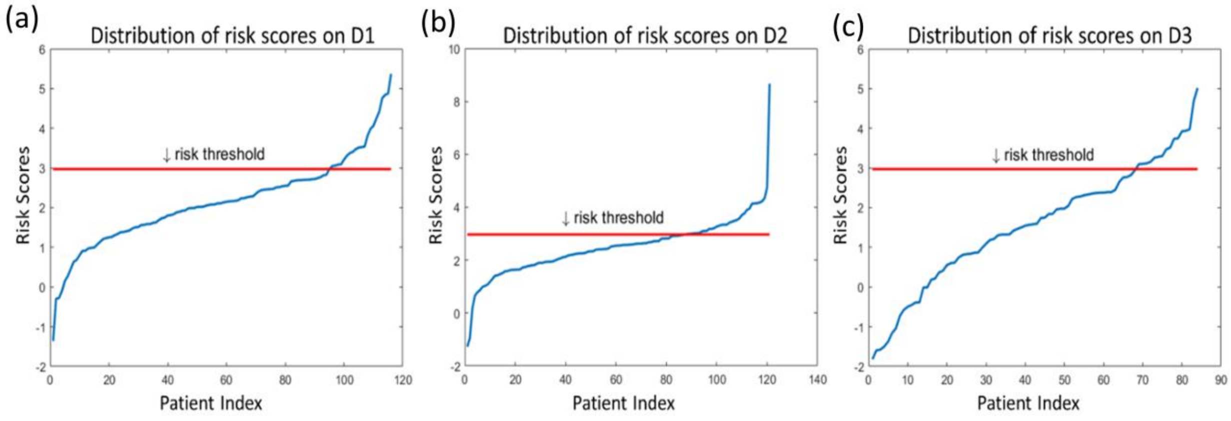


Supplementary figure 3: Distribution of the continuous risk scores on (a) D1, (b) D2, and (c) D3

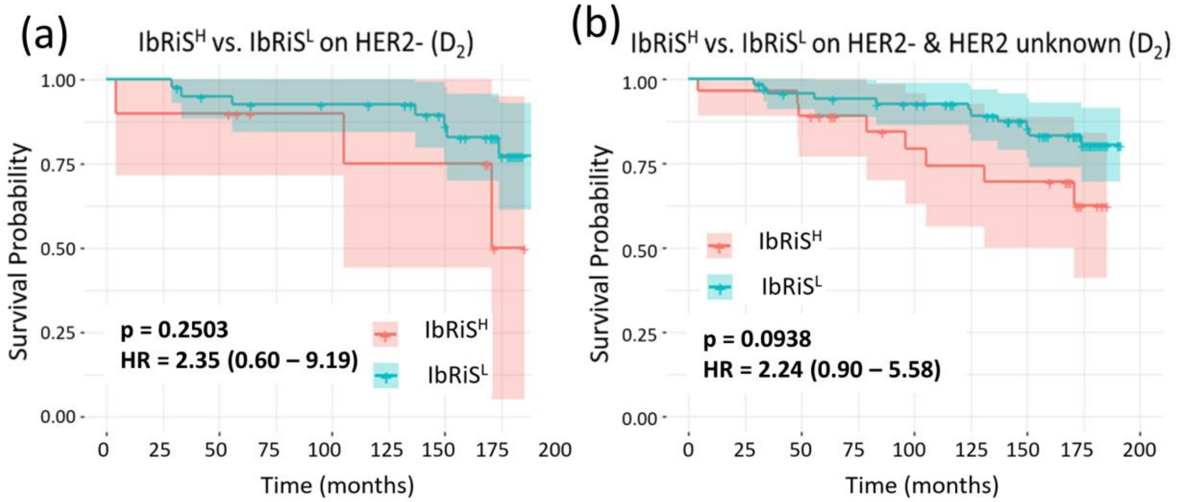


Supplementary figure 4: Prognostic ability of IbRiS on D2 by controlling HER2 status. (a) KM curves illustrate the estimates of DFS on IbRiS$^H$ (red) versus IbRiS$^L$ (blue) on HER2- patients in D2; (b) as well as

IbRiS$^H$ (red) versus IbRiS$^L$ (blue) on the HER2- & HER2 unknown patients in D2 both utilizing two-sided log-rank test to measure the differences between low- and high-risk groups.

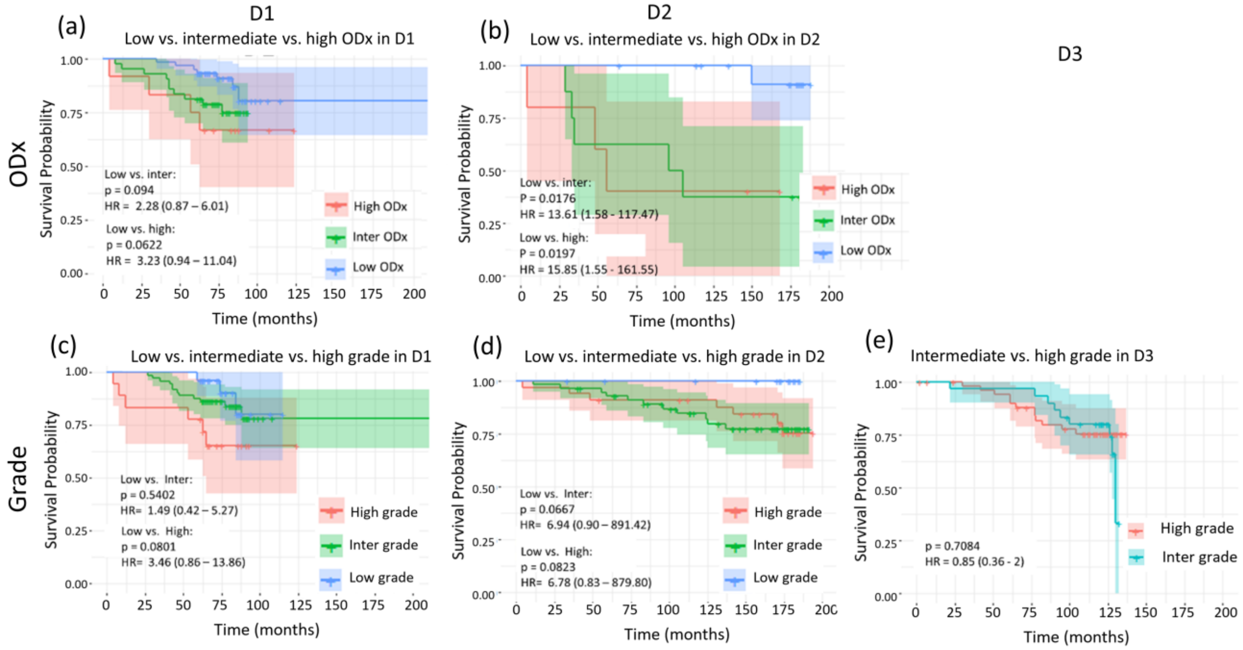


Supplementary figure 5. KM curve estimates for DFS on high versus intermediate versus low ODx risk categories in (a) D1 and (b) D2, and high versus intermediate versus low histologic grade in (c) D1, (d) D2 and (e) D3 with the differences between the risk categories assessed by two-sided log-rank test. ODx risk category was significantly prognostic on D2 but not on D1, while histologic grade was prognostic on neither D1 nor D2.

As Supplementary figure 5 shows, the three ODx risk categories were statistically significantly distinguishable on D2 and, with the low ODx group demonstrating more favorable outcomes than the high/intermediate group. A similar distinguishable trend between the low and high/intermediate ODx risk groups was also exhibited on D1, although not statistically significant. However, the stratification of recurrence risk between intermediate and high ODx groups was ambiguous on both D1 and D2.

The statistically significant risk stratification was neither identified between the low and intermediate histologic grades nor between the low and high histologic grades on any of the three cohorts, although the trend that the high histologic grade had worse outcome could be observed (note only one patient has low histologic grade in D3 and was thus not shown in the KM curves).

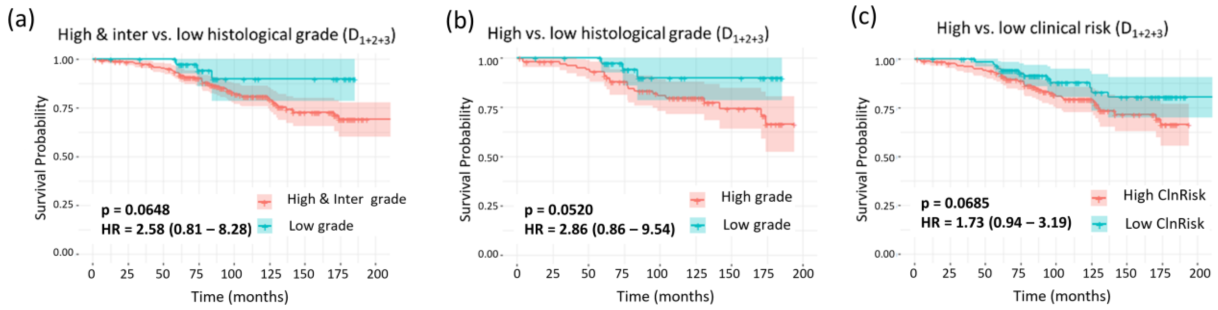

Supplementary figure 6: KM curves estimate the DFS on (a) high & intermediate (red) versus low (blue) histologic grades, (b) high (red) versus intermediate & low (blue) histologic grades, (c) as well as high (red) versus low (blue) clinical risks. The risk stratification was marginally significant both between the high and low histologic grades and between the high and low clinical risk groups as assessed by the two-sided log-rank test.

## Supplementary References


1. MoNuSeg - Grand Challenge. *grand-challenge.org* https://monuseg.grand-challenge.org/Home/.
2. Isola, P., Zhu, J.-Y., Zhou, T. & Efros, A. A. Image-to-Image Translation with Conditional Adversarial Networks. in *2017 IEEE Conference on Computer Vision and Pattern Recognition (CVPR)* 5967–5976 (IEEE, 2017). doi:10.1109/CVPR.2017.632.
3. Ronneberger, O., Fischer, P. & Brox, T. U-Net: Convolutional Networks for Biomedical Image Segmentation. in *Medical Image Computing and Computer-Assisted Intervention – MICCAI 2015* (eds. Navab, N., Hornegger, J., Wells, W. M. & Frangi, A. F.) vol. 9351 234–241 (Springer International Publishing, 2015).
4. Radford, Alec, Luke Metz, and Soumith Chintala. "Unsupervised representation learning with deep convolutional generative adversarial networks." arXiv preprint arXiv:1511.06434 (2015).
5. Salimans, T. *et al.* Improved Techniques for Training GANs. in *Advances in Neural Information Processing Systems* vol. 29 (Curran Associates, Inc., 2016).
6. Mirza, M. & Osindero, S. Conditional Generative Adversarial Nets. *ArXiv14111784 Cs Stat* (2014).
7. Mahmood, F. *et al.* Deep Adversarial Training for Multi-Organ Nuclei Segmentation in Histopathology Images. *IEEE Trans. Med. Imaging* **39**, 3257–3267 (2020).
8. Razavi, S. *et al.* MiNuGAN: Dual Segmentation of Mitoses and Nuclei Using Conditional GANs on Multi-center Breast H&E Images. *J. Pathol. Inform.* **13**, 100002 (2022).
9. Babajide. *Cell Nuclei Segmentation from Histology images using cGAN*. (2023).

10. Janowczyk, A. & Madabhushi, A. Deep learning for digital pathology image analysis: A comprehensive tutorial with selected use cases. *J. Pathol. Inform.* **7**, 29 (2016).

11. Mitosis Counting in Breast Cancer: Object-Level Interobserver Agreement and Comparison to an Automatic Method | PLOS ONE. https://journals.plos.org/plosone/article?id=10.1371/journal.pone.0161286.

12. Zhang, R. *et al.* Reproducibility of the Nottingham modification of the Scarff-Bloom-Richardson histological grading system and the complementary value of Ki-67 to this system. *Chin. Med. J. (Engl.)* **123**, 1976–1982 (2010).

13. Nuclear Shape and Architecture in Benign Fields Predict Biochemical Recurrence in Prostate Cancer Patients Following Radical Prostatectomy: Preliminary Findings - PubMed. https://pubmed.ncbi.nlm.nih.gov/28753763/.

14. Lu, C. *et al.* Nuclear shape and orientation features from H&E images predict survival in early-stage estrogen receptor-positive breast cancers. *Lab. Investig. J. Tech. Methods Pathol.* (2018) doi:10.1038/s41374-018-0095-7.

15. Li, H. *et al.* Quantitative nuclear histomorphometric features are predictive of Oncotype DX risk categories in ductal carcinoma in situ: preliminary findings. *Breast Cancer Res. BCR* **21**, (2019).

16. Haralick, R. M. Statistical and structural approaches to texture. *Proc. IEEE* **67**, 786–804 (1979).

17. Lee, G. *et al.* Cell orientation entropy (COrE): predicting biochemical recurrence from prostate cancer tissue microarrays. *Med. Image Comput. Comput.-Assist. Interv. MICCAI Int. Conf. Med. Image Comput. Comput.-Assist. Interv.* **16**, 396–403 (2013).

18. Ali, S., Veltri, R., Epstein, J. A., Christudass, C. & Madabhushi, A. Cell cluster graph for prediction of biochemical recurrence in prostate cancer patients from tissue microarrays. in vol. 8676 86760H (International Society for Optics and Photonics, 2013).

19. Whitney, J. *et al.* Quantitative nuclear histomorphometry predicts oncotype DX risk categories for early stage ER+ breast cancer. *BMC Cancer* **18**, 610 (2018).

20. Wang, X. *et al.* Prediction of recurrence in early stage non-small cell lung cancer using computer extracted nuclear features from digital H&E images. *Sci. Rep.* **7**, 13543 (2017).

21. Romo-Bucheli, D., Janowczyk, A., Gilmore, H., Romero, E. & Madabhushi, A. A deep learning based strategy for identifying and associating mitotic activity with gene expression derived risk categories in estrogen receptor positive breast cancers. *Cytom. Part J. Int. Soc. Anal. Cytol.* **91**, 566–573 (2017).

22. Elston, C. W. & Ellis, I. O. Pathological prognostic factors in breast cancer. I. The value of histological grade in breast cancer: experience from a large study with long-term follow-up. *Histopathology* **19**, 403–410 (1991).

23. Chang, J. M. *et al.* Back to Basics: Traditional Nottingham Grade Mitotic Counts Alone are Significant in Predicting Survival in Invasive Breast Carcinoma. *Ann. Surg. Oncol.* **22 Suppl 3**, S509-515 (2015).

24. Pantanowitz, L. *et al.* Accuracy and efficiency of an artificial intelligence tool when counting breast mitoses. *Diagn. Pathol.* **15**, 80 (2020).

25. Williams, B., Mandrekar, J. N., Mandrekar, S. J., Cha, S. S. & Furth, A. F. Finding Optimal Cutpoints for Continuous Covariates with Binary and Time-to-Event.

26. Leo, P. *et al.* Computer extracted gland features from H&E predicts prostate cancer recurrence comparably to a genomic companion diagnostic test: a large multi-site study. *NPJ Precis. Oncol.* **5**, 35 (2021).
