# Supplementary material for: Computational pathology improves risk stratification of a multi-gene assay for early stage ER+ breast cancer
Source: NPJ Breast Cancer. 2023 May 17;9:40. doi: 10.1038/s41523-023-00545-y (PMC10192429; doi:10.1038/s41523-023-00545-y)
Supplement: Supplementary file 2 — Reporting Summary [file 41523_2023_545_MOESM2_ESM.pdf]

nature portfolio

Corresponding author(s): Anant Madabhushi

Last updated by author(s): Apr 16, 2023

Reporting Summary

Nature Portfolio wishes to improve the reproducibility of the work that we publish. This form provides structure for consistency and transparency in reporting. For further information on Nature Portfolio policies, see our Editorial Policies and the Editorial Policy Checklist.

Statistics

For all statistical analyses, confirm that the following items are present in the figure legend, table legend, main text, or Methods section.

n/a	Confirmed	
☐	☒	The exact sample size (*n*) for each experimental group/condition, given as a discrete number and unit of measurement
☐	☒	A statement on whether measurements were taken from distinct samples or whether the same sample was measured repeatedly
☐	☒	The statistical test(s) used AND whether they are one- or two-sided<br>*Only common tests should be described solely by name; describe more complex techniques in the Methods section.*
☐	☒	A description of all covariates tested
☐	☒	A description of any assumptions or corrections, such as tests of normality and adjustment for multiple comparisons
☐	☒	A full description of the statistical parameters including central tendency (e.g. means) or other basic estimates (e.g. regression coefficient) AND variation (e.g. standard deviation) or associated estimates of uncertainty (e.g. confidence intervals)
☐	☒	For null hypothesis testing, the test statistic (e.g. *F*, *t*, *r*) with confidence intervals, effect sizes, degrees of freedom and *P* value noted<br>*Give P values as exact values whenever suitable.*
☐	☒	For Bayesian analysis, information on the choice of priors and Markov chain Monte Carlo settings
☐	☒	For hierarchical and complex designs, identification of the appropriate level for tests and full reporting of outcomes
☐	☒	Estimates of effect sizes (e.g. Cohen's *d*, Pearson's *r*), indicating how they were calculated

Our web collection on statistics for biologists contains articles on many of the points above.

Software and code

Policy information about availability of computer code

Data collection	no software was used for data collection
Data analysis	Python3 was used to train and run deep learning models. Handcraft features were extracted using non-published code in matlab 2019.

For manuscripts utilizing custom algorithms or software that are central to the research but not yet described in published literature, software must be made available to editors and reviewers. We strongly encourage code deposition in a community repository (e.g. GitHub). See the Nature Portfolio guidelines for submitting code & software for further information.

Data

Policy information about availability of data

All manuscripts must include a data availability statement. This statement should provide the following information, where applicable:

- Accession codes, unique identifiers, or web links for publicly available datasets
- A description of any restrictions on data availability
- For clinical datasets or third party data, please ensure that the statement adheres to our policy

The datasets used and/or analyzed during the current study are available from the corresponding author on reasonable request.

Human research participants

Policy information about studies involving human research participants and Sex and Gender in Research.

Reporting on sex and gender	The study was focused on women with breast cancer so the only gender involved is female
Population characteristics	The human participants included in this study were female patients with lymph node negative and ER+ breast cancer from US and India.
Recruitment	Training set D1: Breast cancer patients treated between 1996 and 2018 at University Hospitals (UH) having a corresponding ODx score available were identified and retrieved by the pathologists from the hospital archive. The slides were subsequently digitized and transferred. H&E-stained tumor WSI along with clinicopathological/outcome information were available for 519 patients. Patients without any event (recurrence/metastasis/death) were only recruited in this study when at least a five-year follow-up was available. This process resulted in n=116 ER+ & LN- breast cancer patients (n=22 events) in D1. This study was approved by the Institutional Review Board (IRB) at University Hospitals (IRB No 02-13-42C).<br>Validation Cohort D2: D2 comprises subset of patients from ECOG 2197. The access to the ECOG dataset involved a 2-year long process including a proposal review first through ECOG and subsequently through the Cancer Therapy Evaluation Program (CTEP) at the National Cancer Institute (NCI). From this superset, D2 comprises the subset of n=121 ER+ & LN- breast cancer patients (n=23 events), whose corresponding WSIs and clinical information were both accessible. ECOG provided us with the de-identified clinical data from the archived clinical trial along with the de-identified images. . This study was approved by the IRB at University Hospitals (IRB No 02-13-42C).<br>Validation Cohort D3: D3 comprises n=84 ER+ & LN- Indian patients treated in 2009 and with follow-up until 2020 (n=21 events) at Tata Memorial Center (TMC) which were identified and retrieved by the pathologist from hospital archive. The H&E stained tumor slides for individual patients were digitized in and subsequently transferred from TMC. . The study was approved by Institutional Ethics Committee, TMC, IEC no. 1712.
Ethics oversight	The study conformed to HIPAA guidelines was approved by the Institutional Review Board (IRB) at University Hospitals Cleveland Medical Center. IRB No 02-13-42C. The need for written consent from participants was waived due to the use of de-identified retrospective data.

Note that full information on the approval of the study protocol must also be provided in the manuscript.

Field-specific reporting

Please select the one below that is the best fit for your research. If you are not sure, read the appropriate sections before making your selection.

☒ Life sciences ☐ Behavioural & social sciences ☐ Ecological, evolutionary & environmental sciences

For a reference copy of the document with all sections, see nature.com/documents/nr-reporting-summary-flat.pdf

Life sciences study design

All studies must disclose on these points even when the disclosure is negative.

Sample size	Three datasets (N=116, N=121 and N=84) included in this study
Data exclusions	Only patients with lymph node negative and estrogen receptor positive breast cancer were included. The patients with no images or outcome information were excluded. The tissue slides with no tumor present were excluded
Replication	We independently validated our constructed prognostic model from training set on the validation sets to evaluate the robustness of the built model.
Randomization	Our study doesn't involve random allocation of patient samples into different experimental groups. The patients from one institute used as training set and the other two institutes used for validation sets
Blinding	During the image analysis and model construction process, we were blinded to the outcome data for the validation set.

Reporting for specific materials, systems and methods

We require information from authors about some types of materials, experimental systems and methods used in many studies. Here, indicate whether each material, system or method listed is relevant to your study. If you are not sure if a list item applies to your research, read the appropriate section before selecting a response.

Materials & experimental systems

n/a	Involved in the study
☒	☐ Antibodies
☒	☐ Eukaryotic cell lines
☒	☐ Palaeontology and archaeology
☒	☐ Animals and other organisms
☐	☒ Clinical data
☒	☐ Dual use research of concern

Methods

n/a	Involved in the study
☒	☐ ChIP-seq
☒	☐ Flow cytometry
☒	☐ MRI-based neuroimaging

Clinical data

Policy information about clinical studies

All manuscripts should comply with the ICMJE guidelines for publication of clinical research and a completed CONSORT checklist must be included with all submissions.

Clinical trial registration	The study is not a clinical trial
Study protocol	The study is not a clinical trial
Data collection	The study is not a clinical trial
Outcomes	The study is not a clinical trial
